# The development progress of multi-array colourimetric sensors based on the M13 bacteriophage

**DOI:** 10.1186/s40580-022-00351-5

**Published:** 2023-01-03

**Authors:** Sung-Jo Kim, Yujin Lee, Eun Jung Choi, Jong-Min Lee, Kwang Ho Kim, Jin-Woo Oh

**Affiliations:** 1 Bio-IT Fusion Technology Research Institute, Pusan National University, Busan, Republic of Korea; 2 Department of Nano Fusion Technology, Pusan National University, Busan, Republic of Korea; 3 Korea Nanobiotechnology Center, Pusan National University, Busan, Republic of Korea; 4 School of Nano Convergence Technology, Hallym University, Chuncheon, Republic of Korea; 5 Korea and Nano Convergence Technology Center, Hallym University, Chuncheon, Republic of Korea; 6 School of Materials Science and Engineering, Pusan National University, Busan, Republic of Korea; 7 Global Frontier Research and Development Center for Hybrid Interface Materials, Pusan National University, Busan, Republic of Korea; 8 Department of Nanoenergy Engineering and Research Center for Energy Convergence Technology, Pusan National University, Busan, Republic of Korea

**Keywords:** M13 bacteriophage, Colourimetric sensor, Self-templating process, Self-assembly, Genetic engineering, Electronic nose

## Abstract

Techniques for detecting chemicals dispersed at low concentrations in air continue to evolve. These techniques can be applied not only to manage the quality of agricultural products using a post-ripening process but also to establish a safety prevention system by detecting harmful gases and diagnosing diseases. Recently, techniques for rapid response to various chemicals and detection in complex and noisy environments have been developed using M13 bacteriophage-based sensors. In this review, M13 bacteriophage-based multi-array colourimetric sensors for the development of an electronic nose is discussed. The self-templating process was adapted to fabricate a colour band structure consisting of an M13 bacteriophage. To detect diverse target chemicals, the colour band was utilised with wild and genetically engineered M13 bacteriophages to enhance their sensing abilities. Multi-array colourimetric sensors were optimised for application in complex and noisy environments based on simulation and deep learning analysis. The development of a multi-array colourimetric sensor platform based on the M13 bacteriophage is likely to result in significant advances in the detection of various harmful gases and the diagnosis of various diseases based on exhaled gas in the future.

## Introduction

With the development of information and communication technology, the penetration rate of smartphones has increased, and the online market consumption culture using apps has expanded [1]. As consumption pattern analysis becomes easier due to the widespread use of smartphones, distribution is efficiently responding to the consumption culture that has rapidly expanded to the online market during the COVID-19 pandemic. Nevertheless, various imbalances caused by the pandemic have greatly increased the cost of purchasing food essential for survival for the increased world population. To solve this problem, efforts are being made to reduce the losses and costs in the distribution process [2–4]. In the case of fresh foods, such as fruits and vegetables (e.g. climacteric fruits such as tomatoes, bananas, peaches, apples, pears, mangos, and avocados), the loss due to over-ripening during the distribution process is disastrous; therefore, it is highly important to monitor and appropriately control the post-harvest ripening process. The ripening process of fruits and vegetables is related to ethylene which is a plant hormone [5, 6]. The concentration of biosynthetic ethylene at ppm (parts per million) or ppb (parts per billion) levels has a significant effect on the aging process of fresh food [7–12]. Therefore, many studies have been performed to control ethylene-induced ripening [13–15].

Many gas detection strategies have been suggested for monitoring the ripening process. Among these, gas chromatography (GC) is a classical and sensitive detection technique for quantifying gaseous species; the GC consists of an injection port, a column, and a detector [16]. The gaseous mixture is carried by the mobile phase (carrier gas); the mobile phase does not interact with the sample and serves only to transport the gaseous sample throughout the system. A packed or capillary column is used to separate the mixed gases; these columns contain a stationary phase (liquid phase). Each gas component in the mixture is separated as the gaseous mixture passes through the column and interacts with the stationary phase; this causes different mobilities of each gas component. The separated gases are quantified individually using various detectors such as a flame ionisation detector (FID), photoionisation detector (PID), thermal conductivity detector (TCD), and mass spectrometer (MS) [17–19]. GC methods have been used to detect the ripening process of the fruits [20–22].

Other strategies include chemical, electrochemical, and optical approaches. Chemical sensors based on cataluminescene [23], photoluminescence [6, 24, 25], gravimeric [26], and colourimetric detection [27, 28] and electrochemical sensors based on amperometric [29, 30], electrocatalytic [31, 32], and chemoresistive detection have been studied as gas detection techniques [33–36]. For optical sensors, more popular detection methods are often used. Detection techniques based on non-dispersive infrared spectroscopy (NDIR) [37, 38], Raman spectroscopy [39–42], and photoacoustic effects have been used for gas detection [37, 43–45]. Unlike chemical and electrochemical sensors, optical sensors are characterised as non-destructive because they do not rely on chemical reactions for gas detection. These sensors exhibit sensitivities ranging from ppm to ppb. However, these strategies have difficulty responding flexibly to changes in target chemicals in complex and noisy environments.

Recently, an optical sensor using M13 bacteriophage, an eco-friendly biomaterial, has attracted attention for its ability to detect ethylene and other volatile organic compounds (VOCs). Using genetic engineering technology, the intermolecular interactions (or binding affinities) between the M13 bacteriophage and target chemicals can be tuned [46–52]. Because of this tunability, the M13 bacteriophage has been used to develop various gas detectors. Moreover, the M13 bacteriophage interacts with VOC gases without chemical reaction, and thus the sensors based on the M13 bacteriophage exhibit excellent performance for stability and repeatability. Multi-array colourimetric sensors using the M13 bacteriophage have enabled the determination of the origin of agricultural products, detection of explosive chemicals, and diagnoses of lung cancer [53–58]. Based on this principle, multi-array colourimetric sensors have been improved using various types of genetically engineered M13 bacteriophages as electronic nose sensors; these can discriminate complex VOCs mixtures through machine-learning-based on an artificial neural network [55, 56, 59]. Due to its excellent performance and responsiveness to VOCs, colourimetric sensors based on the M13 bacteriophage could enhance research for the detection of various diseases and harmful substances.

In this paper, the recent progress of M13 bacteriophage-based multi-array colourimetric sensors for the development of an electronic nose is reviewed. The main text consists of three parts: (1) Section 2 introduces a self-templating process for fabricating a colour band structure consisting of M13 bacteriophages, (2) Section 3 describes the detection strategies for target chemicals using wild and genetically engineered M13 bacteriophages, and (3) Section 4 reviews how the multi-array colourimetric sensors are optimised for applications in complex and noisy environments. We hope that a better understanding of the electronic nose sensor based on the M13 bacteriophage could advance research for the diagnosis of various diseases and detection techniques for harmful substances.

## M13 bacteriophage-based colour band via a self-templating process

### Colour occurrence of self-assembled structure based on the M13 bacteriophage

**Fig. 1:**
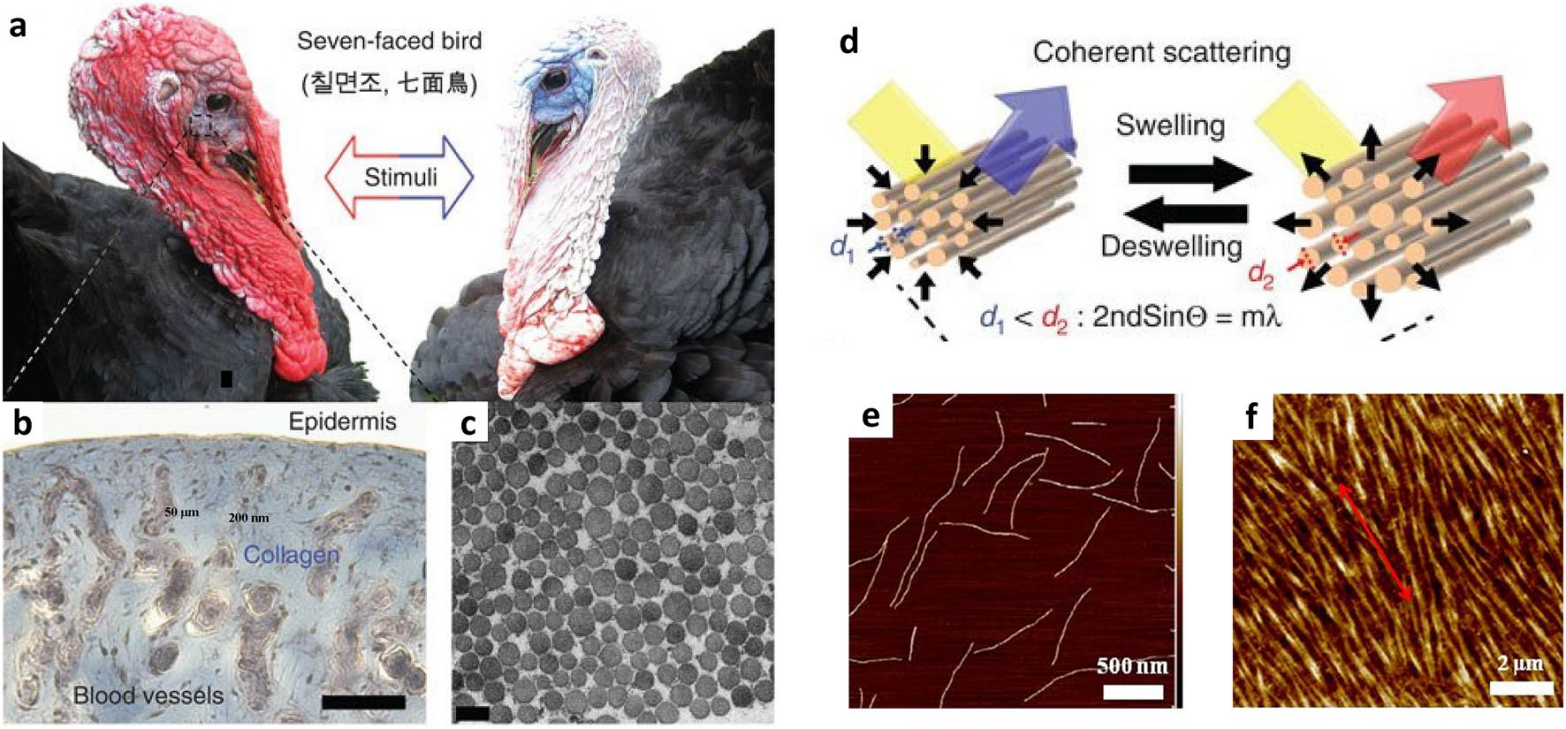
Biomimetic self-assembly and colour occurrence. **a** Colour change of the turkey’s red skin. **b** Collagen and highly vascularized tissues of the turkey skin. **c** Transmission electron micrograph of a cross-section of collagen bundled fibres in the dermis. **d** Coherent scattering of the collagen fibres in turkey skins. AFM images of **e** individual M13 bacteriophages and **f** M13 bacteriophage-based film. The red arrow in **f** represents an orientation of self-assembled M13 bacteriophage bundles. **g** Genetically engineered M13 bacteriophages to recognize target molecules through self-assembled structures (**a**–**d** Reproduced with permission from Ref. [54]. Copyright 2014, Nature Publishing Group. **e**, **f** Reproduced with permission from Ref. [118]. Copyright 2013, Nature Publishing Group)

In nature, many creatures exhibit various colours. Among them, turkey has a very interesting feature in that the skin colour changes according to changes in emotions or stimulation of the surrounding environment (Fig. 1a) [54]. This colour change is due to changes in the collagen nanostructure of the skin regarding the emotion (Fig. 1b, c). A turkey-inspired nanostructure from the M13 bacteriophage-based self-assembly was developed [60]. The distance ($d_1$ and $d_2$) between self-assembled M13 bacteriophage bundles changes regarding external gas exposure resulting in the scattering colour change (Fig. 1d). This colour change happens when the M13 bacteriophage (Fig. 1e) exhibits an ordered thin film (Fig. 1f) by self-assembly represented by a red double-headed arrow. This multi-functionality and easy tunability of self-assembled structure give advantages when we use the M13 bacteriophage as a biomaterial and sensor application.

### Characteristics of the M13 bacteriophage

**Fig. 2:**
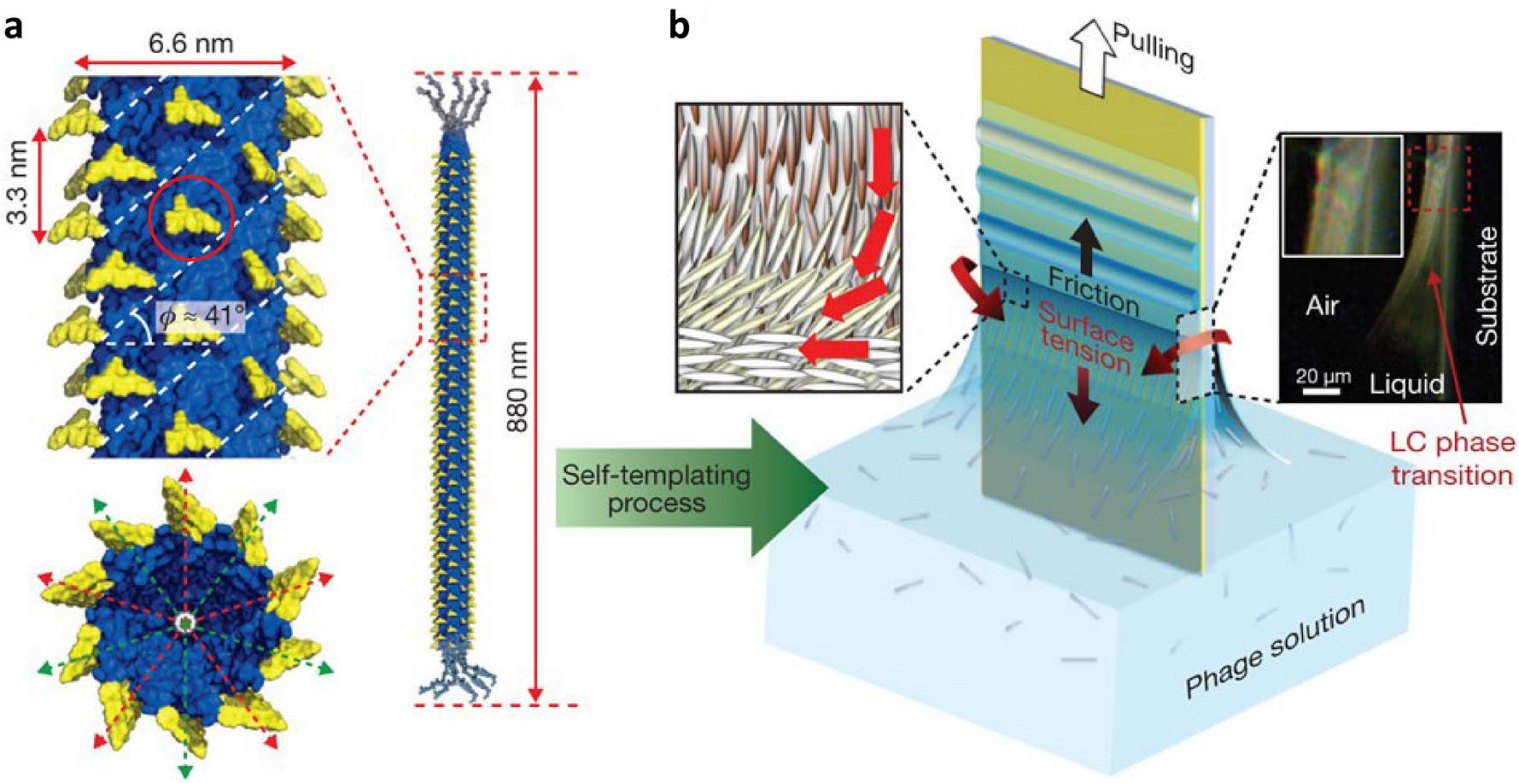
Schematic diagram of the M13 bacteriophage-based autologous template process. **a** Schematic diagram (red and green arrows) of a bacteriophage structure covered with approximately 2700 $\alpha$-helix main capsule (pVIII) protein subunits with five-helix symmetry and a double-thread axis of rotation. The bacteriophage has a helix angle ($\phi$) of $41^{\circ }$ and the periodic subunit spacing is 3.3 nm. The red circle represents the target receptor to recognize target molecules. **b** Schematic of the helical self-template assembly of bacteriophage particles controlled by competing interfacial forces in the meniscus where the liquid crystal phase transition occurs. Polarized light microscopy images show iridescent colours resulting from the formation of liquid crystal phases at the air–liquid–solid interface (**a**, **b** Reproduced with permission from Ref. [60]. Copyright 2011, Nature Publishing Group)

An M13 bacteriophage is flexible; it is a long rod-shaped particle with a 6.6 nm diameter and length of 880 nm (Fig. 2a) [60, 61]. The M13 bacteriophage is covered by approximately 2700 $\alpha$-helical major coat (pVIII) protein subunits with fivefold helical symmetry and a two-fold screw rotation axis, represented by red and green arrows. Here, the helical angle ($\phi$) was $41^{\circ }$ with a periodic subunit spacing of 3.3 nm. The M13 bacteriophage had 4 to 5 pairs of proteins (pIII, pVI, pVII, and pIX) at both ends [61–65]. The self-assembled structures can be tuned because of the surface characteristics of the M13 bacteriophage changed by genetic engineering (red circle of Fig. 2a). The M13 bacteriophage can produce an identical copy of itself through infection of bacterial host cells; this applies to *E. coli*, where the cloned M13 bacteriophages have identical shapes and protein compositions [66–76]. This method takes advantage of the production and purification of the M13 bacteriophage via cell culture. Additionally, a biosensor using the M13 bacteriophage as a bioreceptor would be advantageous in terms of safety because no cases of mutation or harm to the human body have been reported. Some phages similar to M13 bacteriophage obtained Food and Drug Administration (FDA) approval in 2006, demonstrating that they are harmless biocompatible materials [77–79].

Self-assembled thin films consisting of M13 bacteriophages were fabricated using the self-templating method (Fig. 2b). These self-assembled thin films exhibited unique nanostructures and were achieved by controlling the pulling speed of the substrate in solution. The solution refers to the dispersion of the M13 bacteriophages in the solvent. The meniscus due to surface tension occurred at the boundary between the solution and substrate. Water evaporation in the meniscus increased the M13 bacteriophage concentration; this led to a phase change in the solution from isotropic to liquid crystal (LC) phase [80–82]. The LC phase contributes to the fabrication of the well-aligned M13 bacteriophage films on substrates. Besides, the film structures can be adjusted by controlling the pulling speed of the dip coating method.

### M13 bacteriophage-based thin films

**Fig. 3:**
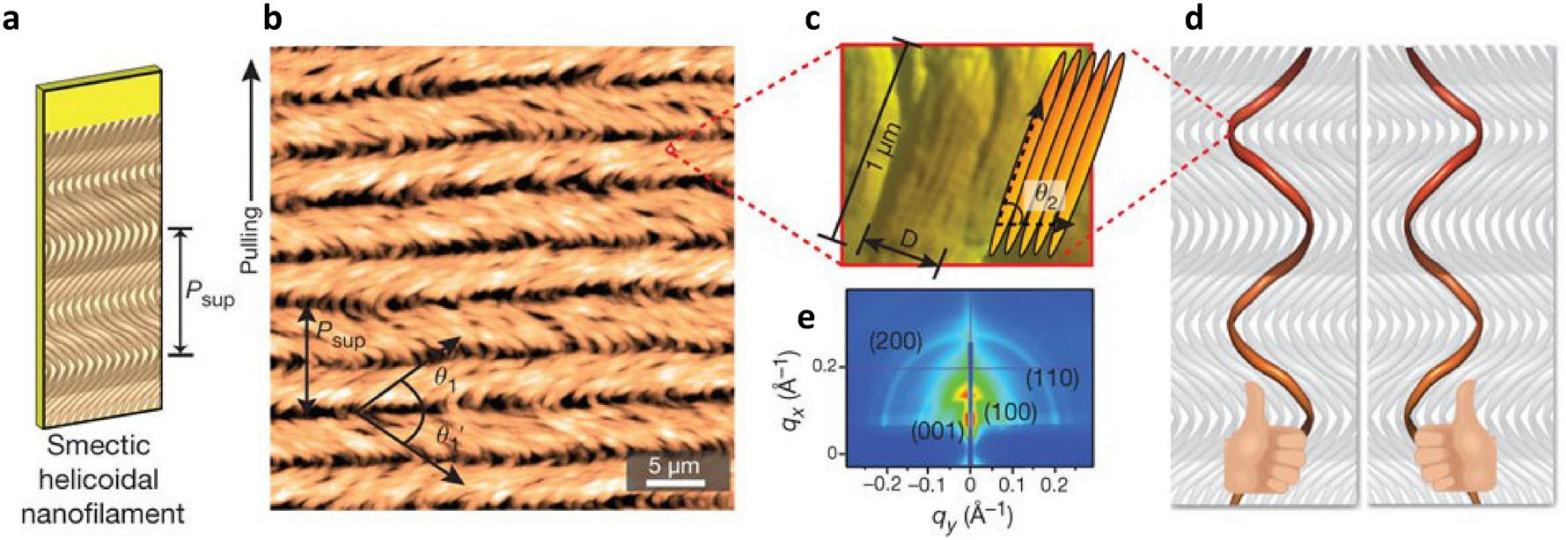
The nanostructure of smectic helicoidal nanofilament. **a** Diagram of smectic helical nanofilament (SHN) structure. Atomic force microscopy (AFM) images of **b** SHN structures composed of **c** smectic C bundles. **d** Proposed model of SHN structure composed of left- and right-handed helix nanofilaments. **e** Grazing incidence small-angle X-ray scattering measurements perpendicular to the pulling direction show phage structures filled with quasi-hexagonal within the SHN (**a**–**e** Reproduced with permission from Ref. [60]. Copyright 2011, Nature Publishing Group)

The dynamic control through this phase change showed various self-assembled nanostructures [60]. The self-templating process resulted in diverse self-assembled structures according to the initial M13 bacteriophage concentration. At a low concentration range (0.1–0.2 mg/mL), M13 bacteriophages formed ordered fibre bundles parallel to the direction of pulling. At a higher concentration range (0.25–1.5 mg/mL), twisted nematic structures were formed at the meniscus; this resulted in the formation of alternating cholesteric-nematic ridges where the grooves have a periodic pitch regarding the stick-slip motion of the meniscus. When the M13 bacteriophage concentration was tuned to 0.25–0.5 mg/mL and the pulling speed to 10–20 $\upmu$m/min, the pinned meniscus contributed to the formation of the cholesteric phase. This pinned meniscus-induced evaporation led to the formation of a cholesteric helical ribbon. In the concentration range of 4–6 mg/mL (Fig. 3a), the deposited films have smectic helical nanofilaments (SHNs); this structure exhibits zigzag morphologies with periodic pitch $P_{\text{sup}}$. The alternating angles of SHN are $\theta _{1}$ and ${\theta_{1}}^{\prime}$ (Fig. 3b). Tilted ($\theta _{2}$) smectic C bundles with fibre diameters were stacked to form the length of each SHN (Fig. 3c). A comparison of the $\theta _{1}$ and ${\theta_{1}}^{\prime}$ values for the left and right sides of the M13 bacteriophage film show $\theta_{1} < {\theta_{1}}^{\prime}$ on the left side, but $\theta _{1} > {\theta_{1}}^{\prime}$ on the right side of the film. The side-dependent differences are indicative of the opposite helical handedness of the nanofilaments caused by the opposing meniscus forces acting from the outer edges towards the centre (Fig. 3d). The grazing incidence small-angle X-ray scattering of SHN (Fig. 3e) showed pseudo-hexagonal packed crystalline structures with (100), (110), and (200) spacing peaks between the M13 bacteriophages.

## Detection methods to respond to diverse target chemicals

### Application to the humidity detecting sensor

**Fig. 4:**
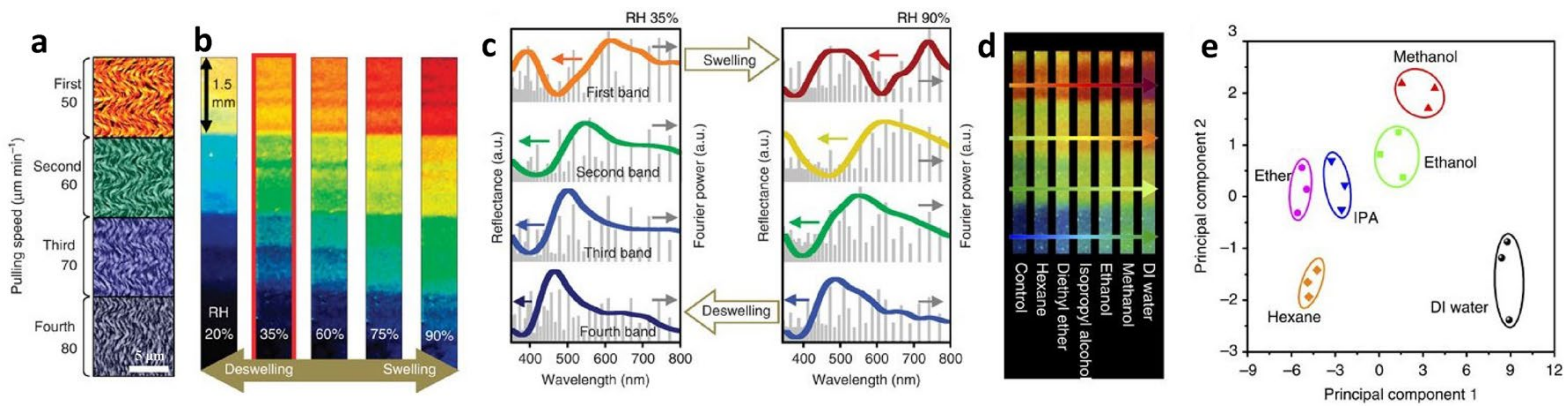
Colour band generation and structural modulation regarding gas exposure. **a** Composite of Atomic force microscopy (AFM) images from different matrices of colour band. Each matrix is composed of quasi-ordered fibre bundles with different diameters and interspacings and exhibits a different colour. **b** A fabricated colour band exhibiting four distinct colours: orange, green, blue, and deep blue (in red box). Changes in relative humidity (RH) result in changes in colours. Depending on the initial phage-bundle structure, each phage matrix swells with a different ratio and exhibits a different colour change. **c** Reflectance spectra (coloured lines) at a normal angle and Fourier power spectra (grey bars) of each phage matrix in 35 and 90% RH; these generally correspond to each other in hue (position of peak) and chroma (shape of the peak; a.u., arbitrary units). **d** Photographs of the colour band after exposure to hexane, diethyl ether, isopropyl alcohol, ethanol, methanol, and DI water. **e** Principal component analysis plot of the colour changes resulting from the exposure of the colour band to different VOCs (**a**–**e** Reproduced with permission from Ref. [54]. Copyright 2014, Nature Publishing Group)

The M13 bacteriophage film constructed with a SHN exhibited selective transmittance/reflectance of visible light similar to a photonic crystal [54, 83, 84]. By controlling the pulling speed (50–80 $\upmu$m/min) of the self-templating process, the M13 bacteriophage film deposited on gold-coated Si wafers exhibited coloured bands without additional concentration control (Fig. 4a) [54]. The alternating angles of zigzag aligned M13 bacteriophage bundles were changed to be approximately $90^{\circ }$ with respect to the pulling speed increment. These exhibited quasi-ordered nanostructure-dependent coloured bands due to coherent light scattering. Since the change in the coloured band depends on the quasi-ordered nanostructures, the relative humidity (RH)-induced swelling and deswelling contribute to the red-shifted and blue-shifted coloured bands, respectively (Fig. 4b). Atomic force microscopy (AFM) images showed that the colour changes were due to the modulation of the M13 bacteriophage bundle structures and subsequent thickness changes; each band swelled to a different extent [54, 60]. When the RH increased from 35 to 90%, the M13 bacteriophage bundle diameter changed by 120, 87, 55, and 32 nm, and the band film thickness changed by 82, 51, 40, and 23 nm for the first, second, third, and fourth matrices, respectively. As shown in Fig. 4c, fast Fourier transform (FFT) analyses of the AFM images further supported that the spectral change of the coloured band was due to swelling and increased interspacing between bundles.

### VOCs detection using colour band structure

The M13 bacteriophage-coloured band showed characteristic colour changes when exposed to volatile organic compounds (VOCs). When the colour band was exposed to VOCs such as hexane, diethyl ether, isopropyl alcohol, ethanol, and methanol, the colour immediately changed (Fig. 4d) [54]. These changes were distinguishable to the naked eye at 300 ppm. The quantitative response of the colour band to the VOCs was recorded using a CCD camera and analysed using the recorded RGB (red, green, and blue) components of the videos. The references were compared with the pattern generated by exposure to an unknown VOC to identify the identity of the samples. Figure 4e shows a two-dimensional (2D) principal component analysis (PCA) plot containing the points for each solvent from three measurements. The two principal components account for 95% of the variance in the measurements. Clustering of data using PCA allowed for the assessment of the discrimination capabilities of the system and demonstrated high reproducibility. This was verified by the clustering of the data obtained for each solvent: deionised (DI) water (p1), methanol (p2), ethanol (p3), isopropyl alcohol (p4), diethyl ether (p5), and hexane (p6). Here, p1 to p6 represent the polarity index. Based on four different colourimetric matrices, the VOCs were discriminated by the polarity index; low-polarity VOCs tended to group together in the negative range of the plot and were well separated from the high-polarity VOCs. The colour change characteristics of the M13 bacteriophage film with respect to the structural change facilitated the development of colour sensors to detect target chemicals using the M13 bacteriophage [46–48, 54].

### Genetic engineering for functional peptide generation on M13 bacteriophage

Genetic engineering provides an opportunity for designing the desired peptide included in the M13 bacteriophage. Peptides can be expressed by regulating the gene for each corresponding protein [85–88]. In particular, the M13 bacteriophage, which specifically binds to the desired substance, can be found using phage display technology [89–91]. It is easy to effectively produce highly efficient functional nanoparticles suitable for this purpose. With these advantageous features, the desired chemical properties can be loaded onto the M13 bacteriophage through genetic manipulation to act as receptors with unique reactivity. To represent reactivity in nature, the bioreceptors were developed by expressing each of the 20 types of amino acids that exist in nature [49–52].

**Fig. 5:**
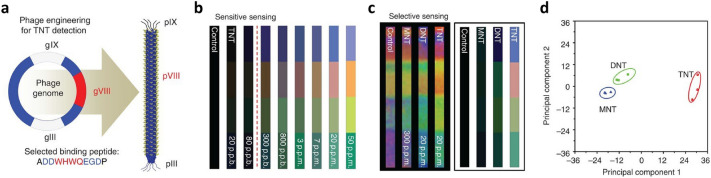
Specific target detection using genetically engineered M13 bacteriophage. **a** 2700 copies of the TNT-binding receptor (WHWQ) identified by directed evolution which are genetically engineered onto the surface of M13 bacteriophage. **b** Detection sensitivity of colour band regarding TNT. The dashed red line indicates the sensitivity limit against TNT. **c** Photos and processed colour fingerprints of colour band after TNT, DNT, and MNT exposure. **d** Principal component analysis (PCA) plot of the colour changes resulting from the exposure of the Phage litmus to TNT, DNT, and MNT (**a**–**d** Reproduced with permission from Ref. [54]. Copyright 2014, Nature Publishing Group)

Figure 5a shows an example of the genetic engineering of the M13 bacteriophage, where the plasmid of the wild-type M13 bacteriophage (Wild-M13 bacteriophage) was tuned to improve the response to explosives. Tuning of the plasmid of wild-M13 bacteriophage led to changes in the 2700 pVIII coat proteins, such as—ADDWHWQEGDP—(WHW-M13 bacteriophage); this change enhanced $\pi$–$\pi$ stacking interaction for aromatic hydrocarbons [54, 60, 92]. The 20 amino acids were expressed as follows: arginine (R), histidine (H), lysine (K), aspartic acid (D), glutamic acid (E), serine (S), threonine (T), asparagine (N), glutamine (Q), cysteine (C), glycine (G), proline (P), alanine (A), valine (V), isoleucine (I), leucine (L), methionine (M), phenylalanine (F), tyrosine (Y), and tryptophan (W).

This colour band consisting of WHW-M13 bacteriophage exhibited an excellent sensitivity to trinitrotoluene (TNT) from the ppm level down to 300 ppb (Fig. 5b). Here, the displayed colour band represents the colour difference ($\Delta \text{RGB}$) from the reference RGB (Red–Green–Blue colour) values. Additionally, the WHW-M13 bacteriophage-based colour band showed selective sensing for mononitrotoluene (MNT), dinitrotoluene (DNT), and TNT; this is shown in Fig. 5c, where the left- and right-handed sides represent the naked colour band and colour difference, respectively. These responses enabled classification using 2-dimensional (2D) principal component analysis (PCA) (Fig. 5d). The change in the colour band based on the genetically engineered M13 bacteriophage was used to measure the explosive and distinguish between MNT, DNT, and TNT. PCA classified two principal components that accounted for 97.2% of the variance in the three measurements of each explosive chemical.

**Fig. 6:**
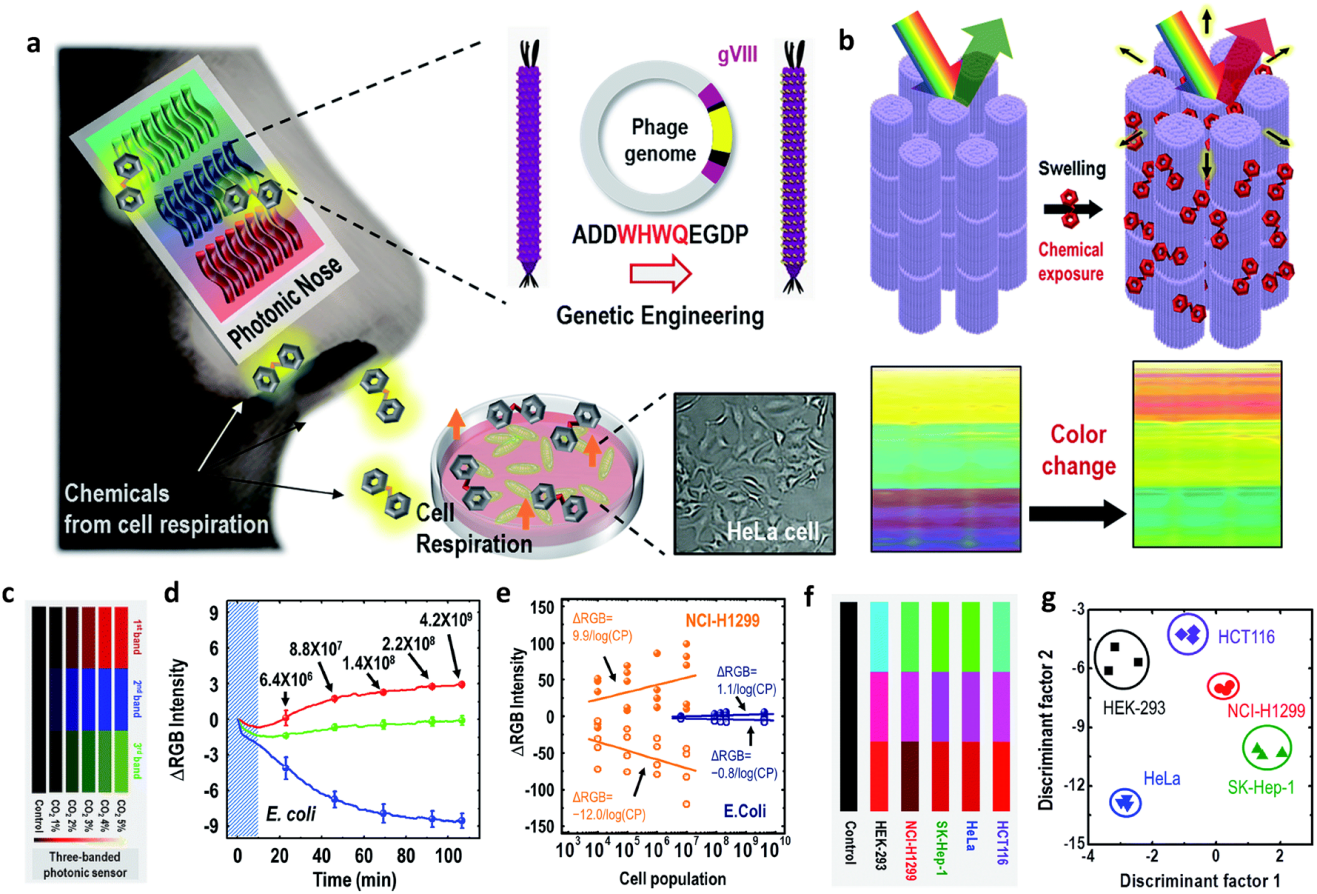
M13 bacteriophage based photonic nose for differential cell recognition. **a** Specific odorants produced by cell breathe. **b** Swelling of M13 bacteriophage bundle responding to vapor phase chemicals resulting in scattering colour change. **c** colour fingerprints from the M13 bacteriophage-based photonic nose on exposure to different carbon dioxide concentrations. **d** Real-time RGB intensity profile for *E. coli* incubated with the photonic nose. **e** RGB intensity change from both *E. coli* and NCI-H1299 cells as a function of different cell populations. **f** RGB colour patterns after exposure to the respiration of different cell types. **g** LDA plot of the colour changes resulting from exposure to HEK-293, NCI-H1299, SKHep-1, HeLa and HCT116 cells (**a**–**g** Reproduced from Ref. [95]. Copyright 2017, The Royal Society of Chemistry)

The WHW-M13 bacteriophage-based colour band was used to detect various other chemicals. A $3\times 3$ multi-array colourimetric sensor (3 colour bands including a 3-colour channel) was utilised to detect differential cell recognition (Fig. 6a). The cells produce specific odorants when they breath. The WHW-M13 bacteriophage-based colour band responds to exposure to the odorants and thus this exhibits colour change (Fig. 6b). The colour sensor detects $\text{CO}_2$ gas (Fig. 6c) generated by *E. coli*. The colour change represents the gas concentration (Fig. 6d). The RGB intensity change of colour sensor can distinguish between both *E. coli* and NCI-H1299 cells (human non-small lung cancer) as a function of different cell populations (Fig. 6e). The colour sensor was tested for toluene, hydrazine, o-xylene, ethanol and ethylbenzene vapors to utilize detecting exhaled gas produced by specific cells. The colour sensor exhibited the RGB patterns (Fig. 6f) and thus this has enabled distinguishing human liver adenocarcinoma (SK-Hep-1), cervical cancer (HeLa), human colon cancer (HCT116), human non-small lung cancer (NCI-H1299), and normal human embryonic kidney (HEK293) (Fig. 6g).

This colour sensor approach was applied to detect various targeted chemical vapours. Benzene derivatives (benzene, toluene, phenol, and chlorobenzene) were detected and discriminated with a sensitivity range of 10–200 ppm. Four phthalate materials (diethyl phthalate, dibutyl phthalate, benzyl-butyl-phthalate, bis-(2-ethylhexyl)-phthalate) and five PCB materials (PCB18, PCB101, PCB138, PCB180, and PCB209) classified as representative endocrine-disrupting chemicals were identified with a sensitivity range of 50–300 ppm [93]. For an application of antibiotic substances, cefadroxil, amoxicillin, and rifampicin were detected at concentrations of 10, 30, 50, and 100 ppm, respectively [94]. Cefadroxil and amoxicillin, which have very similar structures, remarkably showed adequate separation. This sensor has also been used to diagnose diseases [95–99].

Subsequently, a different type of genetically engineered M13 bacteriophage was studied, in which the plasmid was engineered to—AAEEEEDRAKAFN—(4E-M13 bacteriophage), where 2700 pVIII coat proteins of the Wild-M13 bacteriophage were tuned; each pVIII protein has four negatively charged glutamic acid groups at the end, showing a very high-polarity [100]. These characteristics showed a tendency to react very sensitively to the polarity of the target material. Another application involved the detection of foods with complex ingredients [53]. This successfully classified the local origin of domestic agricultural food, such as garlic (94.1%), onion (88.7%), and perilla (91.6%).

### Multi-array colourimetric sensor for enhanced selectivity and sensitivity

**Fig. 7:**
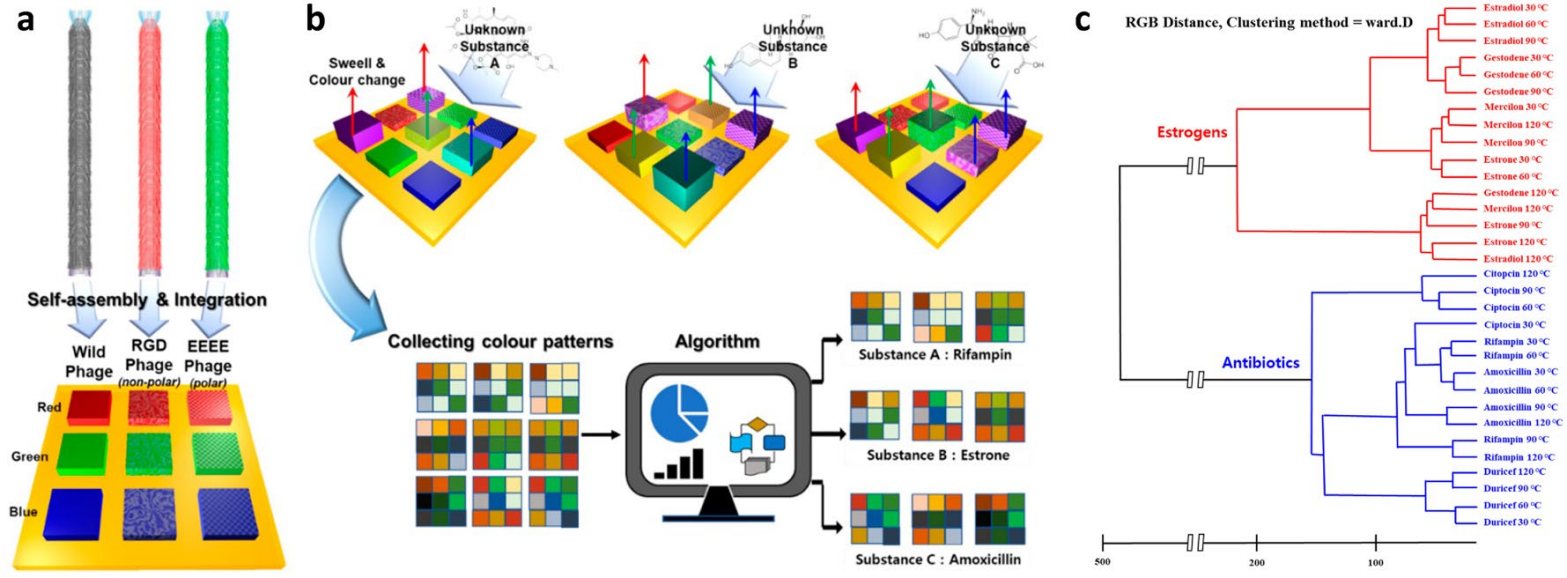
Multi-array colourimetric sensor based on the genetically engineered M13 bacteriophages.** a** Schematic diagram of colourimetric sensor array using genetically engineered M13 bacteriophages. **b** Analytic algorithm to classify an unknown substance. **c** Hierarchical cluster analysis dendrogram for eight types of medical chemicals using the colour distance (Ward.D linkage method) (**a**–**c** Adapted from Ref. [101])

To enhance the detection ability, sensors based on the colour band consisting of the M13 bacteriophage were expanded from a single type to a combined type [101]. The multi-array combined three colour bands consisting of wild-, RGD-, and 4E-M13 bacteriophages (Fig. 7a). The peptide sequence of the RGD-M13 bacteriophage displayed was CGRGDSCGGG [102]. Therefore, each M13 bacteriophage had a different peptide composition for the pVIII protein. This multi-array exhibited a unique colour pattern that was responsible for the target analyte. The RGB of the colour pattern of the target analyte was changed (Fig. 7b); these RGB changes are used for classification. The colour distance (CD) is defined as $\text{CD}=\sum _{i=1}^{n} \sqrt{(R_i^a-R_i^b)^2+(G_i^a-G_i^b)^2+(B_i^a-B_i^b)^2}$ was used for hierarchical clustering analysis (HCA). Where *i* is the index of the multi-array component and *a* and *b* are the colour values before and after exposure to the target analyte, respectively. This detection approach made it possible to classify estrogen-based drugs (Mercilon, gestodene, estrone, and estradiol) and antibiotic-based drugs (Druicef, citopcin, amoxicillin, and rifampin), as shown in the HCA dendrogram in Fig. 7c.

## Optimization strategies of multi-array colourimetric sensors

### Optimization of multi-array colourimetric sensor toward the electronic nose

Dogs smell 10,000 times better than humans, and humans have been conducting sensor research for decades to reach a dog-like sense of smell [103–112]. The electronic nose sensor is a sensor system that monitors the smell coming from the nose of a person and uses the principle of detecting and identifying with the sensor as the smell enters; these use various organic/inorganic receptors as sensing units. To improve the accuracy of the response results of complex materials, PCA and HCA analysis methods were used to classify these materials [101, 113–115]. However, there are some disadvantages in that the diversity of responses is not easily increased and systematic correlations are difficult to determine due to the different characteristics of each receptor. To overcome these disadvantages, an electronic nose sensor was developed based on the characteristics of the M13 bacteriophage.

**Fig. 8:**
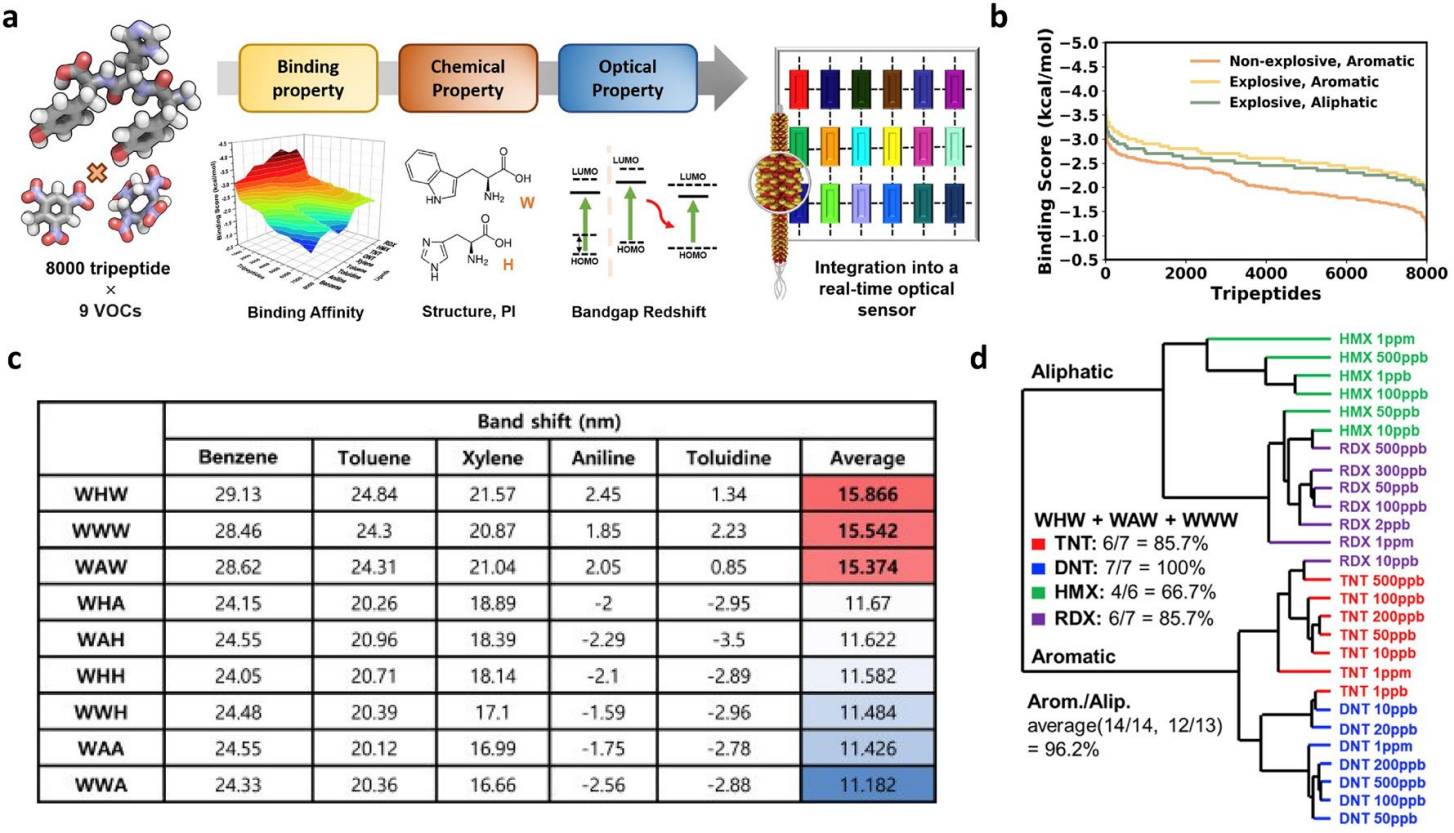
Multi-scale computational prediction and experimental validation. **a** The overall schematization of the screening of 8000 tripeptides versus nine VOCs. **b** Score distribution in the docking of 8000 tripeptide versus 3 chemical classes. **c** Band redshift of functional peptides with W on the N terminus calculated by first-principles simulation. **d** Hierarchical cluster analysis dendrogram of aliphatic and aromatic explosives (**a**–**d** Reproduced with permission from Ref. [55]. Copyright 2021, Elsevier B.V.)

The multi-array colourimetric sensor based on the M13 bacteriophage with genetic engineering has opened a new approach for developing an electronic nose. The first step was to identify an optimised combination of genetically engineered M13 bacteriophages. The last three terminals of the pVIII protein were engineered to alter the intermolecular interactions with the target chemicals. Since 20 amino acids are utilised in genetic engineering, there are 8000 combinations of tripeptides (Fig. 8a) [55]. This was based on the binding affinities from all possible combinations with the VOCs. The binding score was calculated for each molecule, and the peptides were aligned in the order of increasing binding scores. The more negative the binding score, the stronger the binding strength between the peptide and the VOC. Using the calculated binding scores, the database of $8000 \times 9$ arrays enabled the identification of promising peptides; this led to the outstanding performance of our optical biosensor when integrated into the M13 bacteriophage. The binding scores of all 8000 tripeptides in the three chemical classes are shown in Fig. 8b. Explosive compounds exhibited higher bond strengths to tripeptides than their non-explosible aromatic counterparts; this was due to the abundance of electrons in the nitro group and the stronger bonding electrons. Molecular rings without nitro groups lack electrons compared to molecular rings with nitro groups. Therefore, more electrons were driven from the peptide to the ring of the VOC molecule. These properties can be exploited for the selective detection of other target molecules.

The computations indicated that peptides containing W demonstrated a larger red-shifted band than others; the shift became larger as W was placed at the end edge of the peptide (N-terminal) [55]. Figure 8c shows the computed band shifts for the nine functional peptides with W placed on the N terminal. WWW, WHW, and WAW are located at the top three polypeptides; these showed the largest red-shifted band with an average change of more than 15 nm. Despite the two W’s, WWA and WWH showed comparably smaller changes. Based on these results, the WWW, WHW, and WAW were selected for further experimental tests. The HCA (Fig. 8d) was consistent in that aromatic and aliphatic explosives were well classified by optical biosensors. This significantly reduced the size of the electronic nose to nearly half that of the conventional biosensors. An electronic nose equipped with a highly sensitive selective receptor was easy to carry due to the reduction of the small-sized sensing components and high-sensing receptors. This simulation-based optimisation approach for colourimetric sensor development was innovative for saving time and money.

### Practical application of the electronic nose

**Fig. 9:**
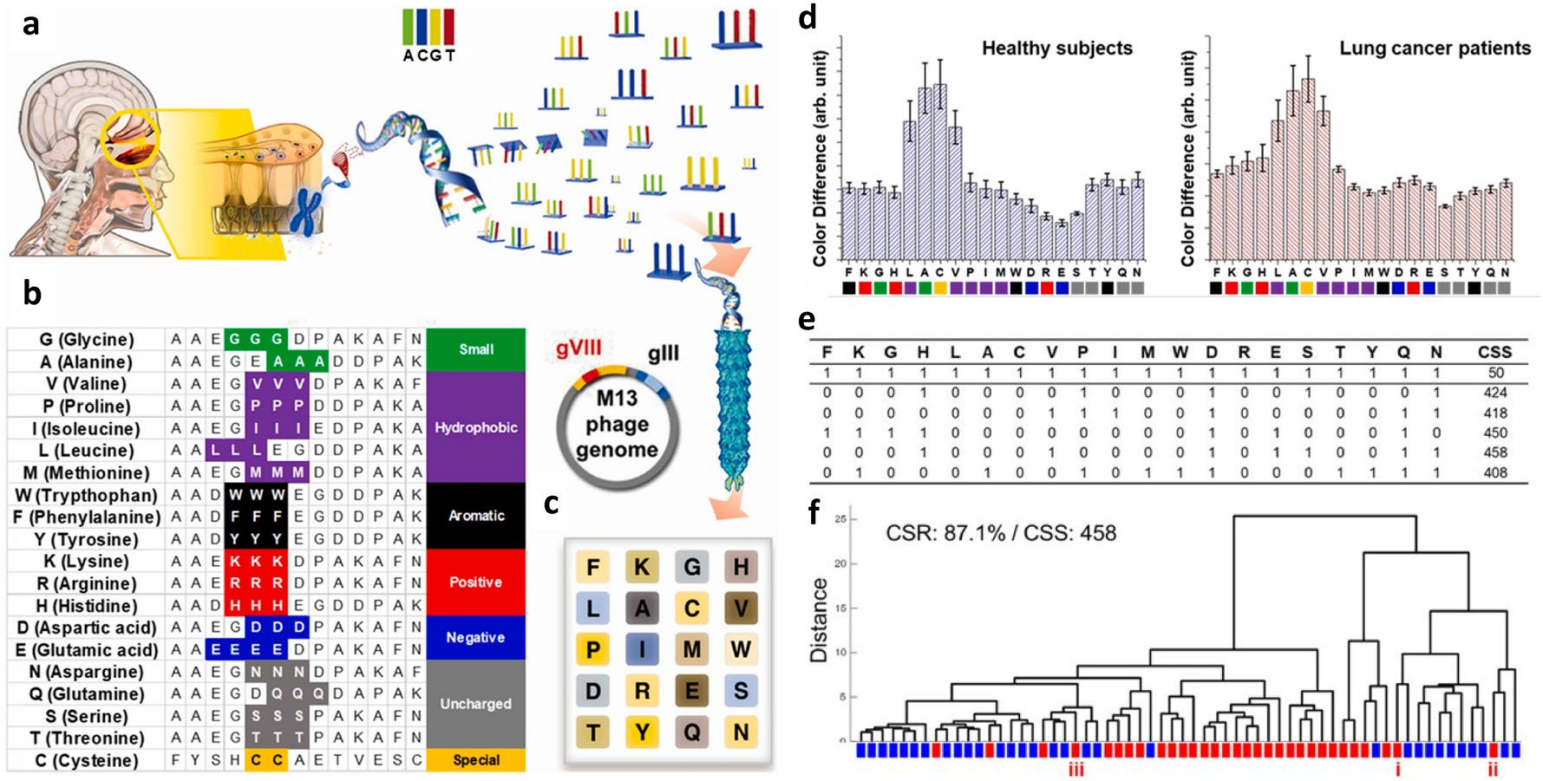
Schematic diagram of the DNA-derived electronic nose. **a** A sequence of 20 amino acids associated with DNA serving as a blueprint for mammalian olfactory receptor cells. Each selected DNA sequence was implanted into a M13 bacteriophage using genetic engineering methods. **b** Sequence listing of genetically engineered M13 bacteriophage. **c** Arrangement of the phage film. **d** Mean colour distance measured by respiration of 31 healthy subjects (left) and 31 patients (right). **e** classification success scores (CSS) histograms calculated for all possible combinations of 20 films. **f** HCA dendrograms based on the best multi-array colourimetric sensor combination selected via neural pattern separation (NPS). Labels i, ii, and iii represent patients with tumours all in the upper lobe and peripheral lesions (**a**–**f** Adapted from Ref. [56])

Even though the efficiency of simulation-based optimisation of the colourimetric sensor was confirmed, there still remains an issue of how to utilise it in practical applications. For example, classifying odours in environments involving different species, including human respiration, is a surprisingly difficult task because it requires the detection of subtle responses to complex and noisy backgrounds. This complex environment makes it difficult to apply simulation-based optimisation methods because they are applied to a single target chemical. Another sensor analysis method was developed to detect chemicals and diseases in complex environments [56]. Twenty genetically engineered phages expressing the reactivity of all mammalian olfactory receptor cells were used for film production (Fig. 9a). The peptide sequence expressed in phage pVIII through genetic manipulation is shown in Fig. 9b. Twenty genetically engineered phages were used to fabricate a $4\times 5$ multi-array colourimetric sensor (Fig. 9c). The colour bands were degraded, and only a single colour domain of colour bands was used for each genetically engineered phage. This multi-array colourimetric sensor was used to diagnose lung cancer during human breathing. Exposure to human breathing caused a colour change and Fig. 9d shows the colour difference between a healthy person (left) and a patient with lung cancer (right). The diagnosis was performed using deep learning analysis with convolutional neural networks (CNNs) and commercial server workstations. The clinical diagnoses of the 31 participating patients confirmed that the characteristics of lung cancer patients were very diverse. However, despite the small size of the data samples and the versatility of their capabilities, the accuracy of the deep learning-based validation exceeded 75.15%.

By introducing neural pattern separation (NPS), which mimics the working principle of the olfactory nerve, it is possible to increase the accuracy of the electronic nose [56]. The NPS method is a computational method used to implant the pattern separation mechanism underlying the memory and learning processes of the mammalian olfactory nervous system into the electronic nose. With the help of NPS (to mimic this pattern separation), the electronic nose learns to respond more sensitively to subtle differences in the respiratory composition. The NPS identifies the sensor elements that are most sensitive to differences between healthy subjects and patients with lung cancer. The classification success scores (CSS) were calculated from the HCA of the 1, 048, 575 dataset ($2^{20}-1$). The top 24 combinations based on the CSS results show that certain sensor unit combinations are effective for classification. The top five CSS combinations are shown in Fig. 9e. Here, a “1” in each digit indicates that the sensor unit corresponding to that location is enabled, and a “0” indicates that it is disabled. When all sensor units are activated, this is expressed as [1 1 1 1 1 1 1 1 1 1 1 1 1 1 1 1 1 1 1 1] and produced a CSS of only 50. The highest CSS of 458 was achieved with [0 0 0 1 0 0 0 1 0 0 0 0 1 0 1 1 0 0 1 1]. The combination of the highest CSS is better for sensor applications.

The classification score ratio (CSR) was re-examined using the combination that received the highest CSS (Fig. 9f). The CSR improved from 75.15 to 87.1%. The phage genotypes (sensor elements) selected were histidine, valine, aspartic acid, glutamic acid, serine, glutamine, and asparagine. The results of the HCA classification using NPS showed a good correlation with the test results of patients with lung cancer. Six patients were misclassified as healthy. In three of these patients, the tumours were all located in the upper lobe, and there was a limitation in respiratory collection due to peripheral lesions. These results show that even among lung cancer patients, various characteristics were mixed; this is an obstacle to HCA and deep-learning-based classification. Only two healthy individuals were incorrectly classified as lung cancer patients. It was difficult to believe that the respiratory characteristics of healthy subjects were similar to those of patients with lung cancer. Therefore, selective electron noses based on NPS suggest that pattern segregation can clarify the distinction between these two groups. These results demonstrate that NPS can be successfully applied in the development of an electronic nose for respiratory-based measurements in humans. This combination selection based on CCS provides an innovative methodology for the development of optimised colourimetric sensors. The characteristics of the M13 bacteriophage-based sensors are summarised in Table 1.

**Table 1:** M13 bacteriophage-based sensors

Object	Method	Phage type	Performance	Refs.
Relative humidity	Spectrum	Wild	20–90%	[54]
Volatile organic compound	PCA	Wild	300 ppm	[54]
	HCA	WWW, HHH	Ripening detection	[116, 117]
Explosive	PCA	WHW	0.3–300 ppm	[54]
	HCA	WHW, WAW, WWW	1 ppb–1 ppm	[55]
Endocrine disrupting chemical	PCA	WHW	10–300 ppm	[93]
Antibiotics	PCA	WHW	10–100 ppm	[94]
Differential cell recognition	PCA	Wild	1–100 ppm	[95]
Origin of agricultural product	SVM	4E	$\sim$ 90%	[53]
Medical chemical	HCA	Wild, RGD, 4E	Diffusion	[101]
Diagnosis	HCA	20 types	Exhaled gas	[56]

Refer that PCA (Principal component analysis), HCA (Hierarchical cluster analysis), and SVM (Support vector machine)

## Conclusions

The M13 bacteriophage-based colourimetric sensors were reviewed. Since the M13 bacteriophage enables modification of its properties utilising genetic engineering, it has been used as a functional building block for self-assembly. A self-templating process was used to fabricate the M13 bacteriophage films [54, 60]. The M13 bacteriophage film exhibited a unique colour because of the coherent light scattering due to the quasi-ordered nanostructures. Among the various nanostructures created by the self-templating process, the smectic helical nanofilaments have been used to develop colourimetric sensors; this is due to the structure being able to control the colour appearance, that is, the colour band. To enhance the detection ability, multi-array colourimetric sensors, which consisted of a genetically engineered M13 bacteriophage, were developed. These M13 bacteriophage-based colourimetric sensors exhibited excellent detection performance for various chemicals in the vapour phase [53–56, 93–95, 101, 116, 117]. The simulation- and machine-learning-based study provided a method for finding the most effective sensor combination among the numerous genetically engineered M13 bacteriophages; this improved the classification rate [55, 56, 59, 117]. This review supports the development of biomaterial-based sensors and the enhancement of electronic nose performance.

## Data Availability

Not applicable.
